# Differential Microbial Pattern Description in Subjects with Autoimmune-Based Thyroid Diseases: A Pilot Study

**DOI:** 10.3390/jpm10040192

**Published:** 2020-10-26

**Authors:** Isabel Cornejo-Pareja, Patricia Ruiz-Limón, Ana M. Gómez-Pérez, María Molina-Vega, Isabel Moreno-Indias, Francisco J. Tinahones

**Affiliations:** 1 Unidad de Gestión Clínica de Endocrinología y Nutrición, Instituto de Investigación Biomédica de Málaga (IBIMA), Hospital Clínico Virgen de la Victoria, 29010 Málaga, Spain; isabelmaria_cornejo@hotmail.com (I.C.-P.); patriciaruizlimon@ibima.eu (P.R.-L.); anamgp86@gmail.com (A.M.G.-P.); molinavegamaria@gmail.com (M.M.-V.); fjtinahones@uma.es (F.J.T.); 2 CIBER Fisiopatología de la Obesidad y Nutrición (CIBEROBN), Instituto de Salud Carlos III, 28029 Madrid, Spain; 3 Department of Medicine and Dermatology, University of Málaga, 29010 Málaga, Spain

**Keywords:** Graves–Basedow’s diseases, Hashimoto’s thyroiditis, autoimmunity, gut microbiota

## Abstract

The interaction between genetic susceptibility, epigenetic, endogenous, and environmental factors play a key role in the initiation and progression of autoimmune thyroid diseases (AITDs). Studies have shown that gut microbiota alterations take part in the development of autoimmune diseases. We have investigated the possible relationship between gut microbiota composition and the most frequent AITDs. A total of nine Hashimoto’s thyroiditis (HT), nine Graves–Basedow’s disease (GD), and 11 otherwise healthy donors (HDs) were evaluated. 16S rRNA pyrosequencing and bioinformatics analysis by Quantitative Insights into Microbial Ecology and Phylogenetic Investigation of Communities by Reconstruction of Unobserved States (PICRUSt) were used to analyze the gut microbiota. Beta diversity analysis showed that gut microbiota from our groups was different. We observed an increase in bacterial richness in HT and a lower evenness in GD in comparison to the HDs. GD showed a significant increase of *Fusobacteriaceae*, *Fusobacterium* and *Sutterella* compared to HDs and the core microbiome features showed that *Prevotellaceae* and *Prevotella* characterized this group. *Victivallaceae* was increased in HT and was part of their core microbiome. *Streptococcaceae*, *Streptococcus* and *Rikenellaceae* were greater in HT compared to GD. Core microbiome features of HT were represented by *Streptococcus*, *Alistipes*, *Anaerostipes*, *Dorea* and *Haemophilus*. *Faecalibacterium* decreased in both AITDs compared to HDs. PICRUSt analysis demonstrated enrichment in the xenobiotics degradation, metabolism, and the metabolism of cofactors and vitamins in GD patients compared to HDs. Moreover, correlation studies showed that some bacteria were widely correlated with autoimmunity parameters. A prediction model evaluated a possible relationship between predominant concrete bacteria such as an unclassified genus of *Ruminococcaceae*, *Sutterella* and *Faecalibacterium* in AITDs. AITD patients present altered gut microbiota compared to HDs. These alterations could be related to the immune system development in AITD patients and the loss of tolerance to self-antigens.

## 1. Introduction

Autoimmune thyroid diseases (AITDs) are the most common organ-specific autoimmune disorders. Within AITDs, Hashimoto’s thyroiditis and Graves–Basedow’s disease are the most frequent conditions.

Hashimoto’s thyroiditis (HT) is identified by lymphocytes infiltration in the thyroid gland which leads to the destruction of thyroid follicles, and the production of autoantibodies against thyroid peroxidase (TPO, 90–95%) and thyroglobulin (TG, 20–50%) [1,2].

On the other hand, Graves’ disease (GD) is identified by the production of autoantibodies of the immunoglobulin G1 subclass that are directed against the thyrotropin receptor which induces thyroid hormone overproduction and also causing hypertrophy and hyperplasia of thyroid cells [3].

The etiology of AITDs is thought to be multifactorial, arising from an interaction between genetic susceptibility, epigenetic, and various endogenous and environmental factors [4].

Several studies have provided evidence for genetic factors, however, the concordance rate for AITDs among monozygotic twins is in the range of 35–55% compared with 3% in dizygotic twins, emphasizing that other important factors, such as the environment, are implicated in the pathogenesis of AITD [5,6,7].

Gut microbiota takes part in the homeostasis of the host, and especially in the regulation of the immune system. Gut microbiota is a promising agent for the development of personalized medicine as it collects information about its host, like diet or environmental factors [8]. Recent evidence suggest that the alteration of gut microbiota may have connection with autoimmune diseases [9,10,11,12].

Notwithstanding, the pathogenic link between gut microbiota and AITDs has not been fully elucidated, with only a few studies in humans [13,14,15]. Zhao et al., demonstrated that HT patients had an altered gut microbiota profile compared with the healthy population and this profile correlated with clinical parameters [16].

In the present study, we aimed to investigate the gut microbiota profile in patients affected by AITD, both HT and GD; and its relationship with autoimmunity markers.

## 2. Materials and Methods

### 2.1. Participants

A total of 18 AITD patients (nine GD patients and nine HT patients) were recruited at the Department of Endocrinology and Nutrition of Virgen de la Victoria University Hospital (Málaga, Spain). Moreover, we included 11 otherwise healthy donors (HDs) with similar anthropometric features as study groups, without thyroid disease and without family history of thyroid disease. These HDs were euthyroid and goiter was not evident on physical examination. Individuals were included from those who were attended at the hospital during the first half of 2018.

All participants with GD were receiving synthetic antithyroid (Neo-tomizol, 5–20 mg of dose) and HT patients were receiving levothyroxine treatment (Eutirox, 50–175 µg of dose) and presented an acceptable control of the disease which allowed us to focus on the pathophysiology of autoimmunity and not on thyroid dysfunction. The exclusion criteria were: pregnancy; type 1 or type 2 diabetes mellitus; other autoimmune disease; and chronic and/or severe gastrointestinal disorders. We also excluded patients and HDs with extreme diets, those exposed to antibiotic therapy (current or previous 3 months), chronic drugs different to AITDs medication that alter microbiota profile, those taking probiotic agents, and the non-acceptance of informed consent.

The study protocol was approved by the Medical Ethics Committee of Virgen de la Victoria University Hospital and conducted according to principals of the Declaration of Helsinki. All participants enrolled provided their written informed consent and were also verbally informed of the characteristics of the study.

### 2.2. Anthropometric and Laboratory Measurements

Anthropometric measurements, including body weight, height, and waist and hip circumferences, were collected. Peripheral venous blood samples were obtained after 8 hours of fasting. The serum was centrifuged at 4000 rpm for 15 min at 4 °C and frozen at −80 °C until analysis. Levels of cholesterol, triglycerides, HDL-cholesterol, and glucose were analyzed by enzymatic methods (Randox Laboratories Ltd., Crumlin, UK) and glycosylated hemoglobin (HbA1c) was determined by Dimension Vista autoanalyzer (Siemens Healthcare Diagnostics, Munich, Germany).

Laboratory markers of autoimmunity [thyroid stimulating immunoglobulin (TSI), anti- thyroperoxidase (Anti-TPO)] and thyroid profile [thyroid-stimulating hormone (TSH), free thyroxine (FT4), free triiodothyronine (FT3)] were quantified as part of the routine patient management in the case of AITD patients, with exception of HDs. Reference ranges were for TSH 0.4–5 μIU/mL; FT4 11–22 pmol/L; and FT3 2–5 pmol/L. TSI was measured by Elecsys Anti-TSHR test following the manufacturer’s protocols (Roche, Basel, Switzerland) in a Roche electrochemiluminometric analyzer (Cobas e 801, Roche, Basel, Switzerland). Anti-TPO was detected by Atellica IM Anti-Thyroid Peroxidase assay (SIEMENS Healthineers, Erlangen, Germany) using the Atellica IM Analyzer (SIEMENS Healthineers, Erlangen, Germany). Reference ranges were for TPO-Ab > 60 IU/mL and TSI-Ab > 2 IU/mL.

### 2.3. DNA Extraction from Faecal Samples

Faecal samples were obtained by the volunteers, immediately refrigerated, and carried to the laboratory, where they were stored at −80 °C for subsequent analysis.

DNA was extracted from 200 mg of stool using the QIAamp DNA stool Mini kit (Qiagen, Hilden, Germany) according to the manufacturer’s protocols. DNA concentration was measured by absorbance at 260 nm, and the purity was verified by determining the A260/A280 ratio with a Nanodrop spectrophotometer (Nanodrop Technologies, Wilmington, DE, USA).

### 2.4. Sequencing of 16S rRNA and Bioinformatic Analysis

The Ion 16S Metagenomics Kit (Thermo-Fisher Scientific Inc., Waltham, MA, USA) was used to amplified the ribosomal 16S rRNA gene region from stool DNA, with two primer sets (V2–4–8 and V3–6, 7–9) covering the most of the hypervariable regions of the 16S rRNA region in bacteria. The libraries were created using the Ion Plus Fragment Library Kit (Thermo Fisher Scientific, Waltham, MA, USA). Barcodes were added to each sample using the Ion Xpress Barcode Adapters kit (Thermo Fisher Scientific, Waltham, MA, USA). Emulsion PCR and sequencing of the amplicon libraries were carried out on an Ion 520 chip (Ion 520TM Chip Kit) using the Ion Chef System and Torrent S5TM system, respectively, using the Ion 520TM/530TM Kit-Chef (Thermo Fisher Scientific, Waltham, MA, USA) according to the manufacturer’s instructions. Torrent Suite™ Server software (Thermo Fisher Scientific, Waltham, MA, USA), version 5.4.0, with default parameters for the 16S Target Sequencing (bead loading ≤30, key signal ≤30, and usable sequences ≤30) was used to base calling and run demultiplexing. Raw data is stored at the public repository SRA database (NCBI) with the BioProject PRJNA666641.

The open-source Quantitative Insights into Microbial Ecology (QIIME2, version 2019.7) software was used to analyze the quality sequences [17,18] and also was used for diversity analysis and subsequent taxonomic analysis through clustering with search [19] and the reference base Greengenes version 13_8 at 97% of identity.

Metagenome function was predicted by Phylogenetic Investigation of Communities by Reconstruction of Unobserved States (PICRUSt) analysis through picking operational taxonomic units (OTUs) from the Greengenes database, as described elsewhere [20]. The resulting OTU table was employed to predict the metagenome at three different Kyoto Encyclopedia of Genes and Genomes (KEGG) Orthology (KO) levels (level (L) 1 to L3).

### 2.5. Statistical Analysis

The open-source Statistical Analysis of Metagenomic Profiles [STAMP (v 2.1.3)] [21] was used to compare the differential abundances of taxa, KEGG categories, and subcategories, with the White’s non-parametric test. α- and β-diversities were analyzed within QIIME2, through the diversity plugin: β-diversity metrics employed a permutational multivariate analysis of variance (PERMANOVA) with 999 permutations, while α-diversity metrics involved a Kruskal–Wallis test.

Anthropometric and clinical characteristics were analyzed with the IBM SPSS Statistics 25 (IBM, Armonk, NY, USA). The relationship between gut microbiota and thyroid variables was analyzed using Spearman´s correlations models. Binary logistic regression models were fitted to assess the relationship between thyroid profile and autoimmunity markers with particular features as independent predictors. To control for potential confounding factors, their results were adjusted by age (years), sex (male or female) and body mass index (BMI, kg/m^2^). The results were described as mean and dispersion (standard deviation, SD) for quantitative variables and as a proportion for qualitative variables. Statistical significance was established at *p* < 0.05. *p* values were corrected for multiple comparisons using the Benjamini–Hochberg method at 0.1, and reported as q-value, when appropriate.

## 3. Results

### 3.1. Clinical Data Study

All subjects (*n* = 29) were of Spanish nationality and born and grown in the Andalusian community. The study groups showed similar clinical characteristics about age, sex, anthropometric parameters, or metabolic profile, without finding statistically significant differences (*p* > 0.05). Our AITD patients had normalized thyroid function by medical treatment, substitution with levothyroxine in HT patients, or antithyroid drugs in patients with GD. However, we found significant differences in autoimmunity between the three study groups. TPO-Ab was significantly increased in HT and TSI-Ab was significantly increased in GD. HDs values of TPO-Ab resulted in slightly increased levels than expected for healthy individuals. In addition, according to serum TSH range our HDs were euthyroid. These demographic and clinical data of the subjects are summarized in Table 1.

### 3.2. Gut Microbiota Diversity in AITD Patients

**Figure 1:**
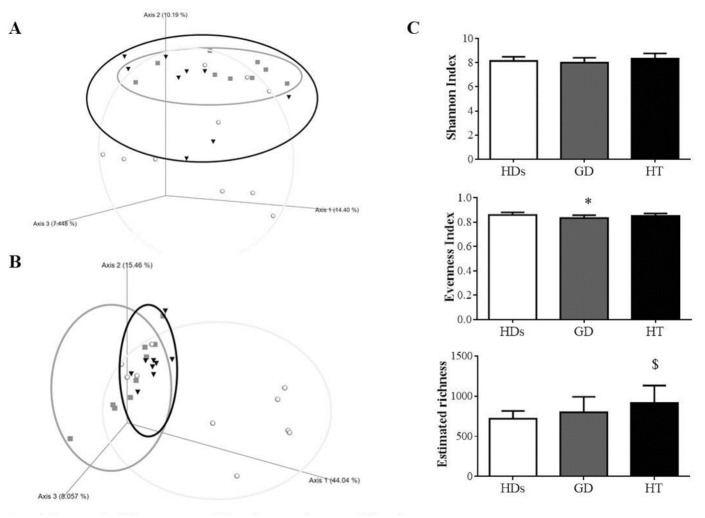
Estimation of diversity in healthy donors (HDs), Graves–Basedow’s disease (GD) patients, and Hashimoto’s thyroiditis (HT) patients. Clustering of fecal bacterial communities according to the different study groups by PCoA using unweighted and weighted UniFrac distances. Statistical differences were observed between groups. (**A**) Unweighted UniFrac distances, *p* = 0.003; and (**B**) Weighted UniFrac distances, *p* = 0.002. Dots belong to the HDs group; square to GD patients and cone to HT patients. (**C**) Shannon Diversity and Evenness indexes and estimated richness among different groups were compared. All values are mean ± SD. * *p* < 0.05 GD patients vs. HDs; $ *p* < 0.05 HT patients vs. HDs. Circles belong to the HDs; squares to GD patients and triangles to HT patients.

After the quality assessment, a total of 2,785,614 quality 16S rRNA gene sequences with an average of 26,056 ± 29,024 sequences per sample passed the filters. In order to compare the populations of the different groups, dimensional Principal Coordinates Analysis plots of UniFrac distances were used. Qualitative (Unweighted UniFrac distance) and quantitative (Weighted Unifrac distance) relationships showed differences among the groups (*p* = 0.003 and *p* = 0.002, respectively) (Figure 1A,B). Further analysis showed that our AITD patients did not show significant differences (*p* > 0.05), but they showed statistically significant differences with respect to HDs (*p* < 0.05 in groups, AITD patients related to HDs) (Figure S1).

Alpha diversity was assessed using rarefaction curves. Richness, estimated by the observed features index, demonstrated a significant increase in HT patients versus HDs (*p* = 0.022), while no differences were found between the other groups. Evenness, calculated by the Pielou index, showed a significant decreased in GD patients compared with HDs (*p* = 0.03) and no significant differences among the other groups. Finally, biodiversity, estimated by the Shannon index, suggested no significant differences among the study groups (Figure 1C).

### 3.3. Gut Microbiota Profile in AITD Patients

**Figure 2:**
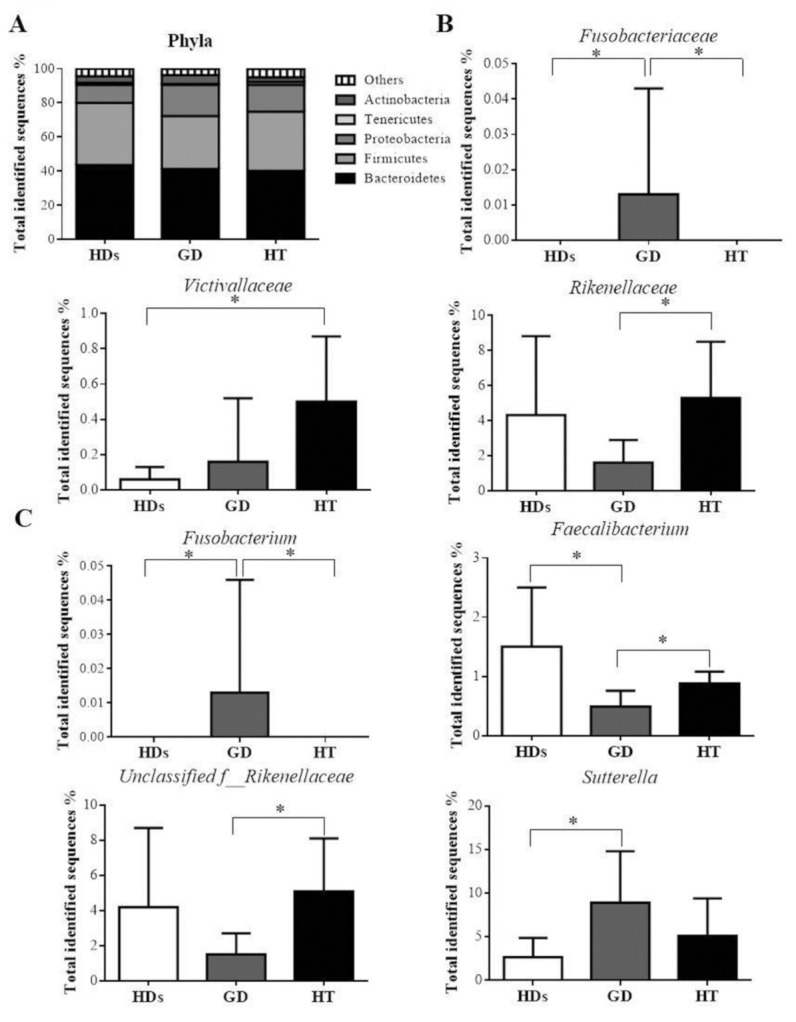
(**A**) Microbiota profile of faecal samples from the study groups at the phylum level. (**B**) Families statistically significant between the study groups. (**C**) Genera statistically significant between the study groups. * Indicates significant differences between groups, q < 0.1 (q = *p*-FDR-corrected).

Dominant bacterial phyla were Bacteroidetes and Firmicutes, while Proteobacteria, Actinobacteria, and Tenericutes shared smaller proportions, between 1–10%, in the different study groups. No differences were observed between our study groups at this taxa level (Figure 2A).

At the family level, *Fusobacteriaceae* was significantly increased in GD patients when compared with HT patients and HDs (q = 0.01). Furthermore, a significantly higher abundance was found in HT patients when compared with HDs for *Victivallaceae* (q = 0.03). In addition, *Rikenellaceae* was significantly decreased in GD patients compared with HT patients (q = 0.04) (Figure 2B).

Finally, at the genus level, *Fusobacterium* was significantly higher in GD patients compared with HDs and also compared with HT patients (q = 0.02 and q = 0.009, respectively). *Faecalibacterium* was significantly lower in GD patients compared to HDs and also compared with HT patients (q = 0.02 and q = 0.07, respectively). An unclassified genus of the family *Rikenellaceae* was significantly lower in GD patients compared to HT patients (q = 0.03). The genus *Sutterella* was significantly higher in GD patients compared to HDs (q = 0.10) (Figure 2C).

### 3.4. Core Microbiome in AITD Patients and HDs

**Figure 3:**
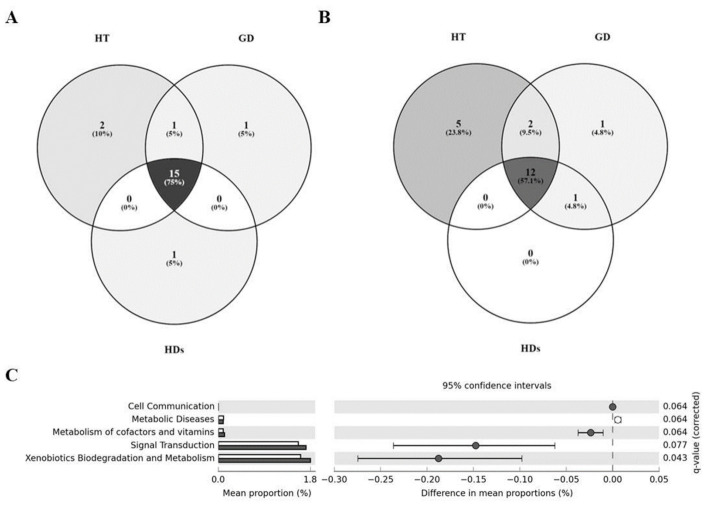
(**A**) Venn diagram of the core microbiome from the study groups at the family level. (**B**) Venn diagram of the core microbiome from the study groups at the genus level. (**C**) Significant differences in predicted functional composition at level 2 of Kyoto Encyclopedia of Genes and Genomes (KEGG) Pathways of the gut microbiota among HDs (white) and GD patients (dark grey). Only functional capacities with *p* < 0.1 are shown; q = *p*-value FDR-corrected.

After studying the differences between groups, we wondered if each group was characterized by a concrete core gut microbiome. We investigated the core microbiome of each group, meaning those features that were shared among the 85% of the samples of each study group. At the family level, we observed that a total of 15 identified families were shared by all the volunteers of the study (See the complete list in Table S1). Interestingly, *Pasteurellaceae* was only shared between AITD patients. *Victivallaceae* and *Streptococcaceae* were characteristic of the HT group and *Prevotellaceae* of GD group; while *Christensenellaceae* seemed to be characteristic of the HDs group (Figure 3A). On the other hand, 12 identified genera were shared between the three groups (See the complete list in Table S1). *Collinsella* and *Roseburia* belonged to the core microbiome of both AITDs, while *Butyricimonas* was shared by GD patients and HDs. HT patients were the group with a greater number of characteristic features: *Streptococcus*, *Alistipes*, *Anaerostipes*, *Dorea* and *Haemophilus*; while *Prevotella* was characteristic of the GD group. No characteristic genus was found in the HDs group (Figure 3B).

### 3.5. Differences in the Metabolic Profiles of Gut Microbiota between AITD Patients and HDs

PICRUSt metagenome predictions were used to identify those microbial functions that were enriched or degraded in AITD patients and HDs. In tier 2 of the KO categories, we observed no significant differences between HT patients and HDs or between both pathologies. However, we found pathways significantly different between GD patients and HDs at this level (Figure 3C).

Moreover, in tier 3 of the KO categories, we have not observed changes between HT patients and HDs. Nevertheless, we have detected a high number of pathways significantly different between GD patients and HDs at this level. The 27 pathways can be observed in detail in Figure S2.

### 3.6. AITDs Could Be Related to Bacterial Profile

Correlation studies were performed to establish a relationship between the thyroid autoimmunity of the study groups and their gut microbiota. Significant univariate correlations were found between the number of specific bacteria with thyroid autoimmunity markers like thyroperoxidase antibody (TPO-ab) (positive correlation with *Alistipes*; *Ruminococcaceae* unclassified and *Enterobacteriaceae*, so negative correlation with *Faecalibacterium*) and thyroid stimulating immunoglobulin antibody (TSI-ab) (positive correlation with *Lactobacillus* and *Pasteurellaceae* and negative correlation with *Faecalibacterium*) (Table 2).

When we evaluated the possible implication of the gut microbial community in global AITDs, we found that an unclassified genus of *Ruminococcaceae* was associated with a 49.4% rise in the odds of the TPO-ab presence in the crude model. This trend was also maintained in the adjusted model by BMI, age, and sex (Table 3). Likewise, we found that *Sutterella* could influence in TSI-ab presence, with a 35.1% increase in either the crude model or the adjusted model. However, *Faecalibacterium* was associated with a decrease of 94.1% in the odds of presenting positive TSI autoimmunity in the crude model, so this relationship is maintained in the adjusted model (Table 4).

## 4. Discussion

Our study has pointed out that the AITDs share common as well as particular gut microbiome profile features that differ from the stool profile of HDs and those dysbiotic amounts of particular taxa could be related with the development of the disease.

Autoimmune disease initiation has been linked to gut microbiota dysbiosis by different mechanisms such as molecular mimicry, the by-stander activation, and the epitope spreading. In recent years, patients bearing with an already established AITDs have also been reported to show alterations of the gut microbial composition [22,23,24].

Gut microbiome profiles from our study groups are different according to beta diversity analysis. However, further comparisons showed that the main differences belonged to the microbiota populations of HDs and each one of the AITDs studied. Thus, AITDs could share some patterns of their microbiota profiles. AITDs microbial changes have been classically attributed to morphological changes. Interestingly, patients with HT presented ultrastructural morphological changes of distal duodenum enterocytes, a variation in microvillus thickness, and a leaky gut condition [22]. On the other hand, GD frequently impacts the hollow organs with lower levels of gastric acid production, increased the intestinal motility which along with autoimmune gastritis contributing to diarrhea. Thus, GD might reshape the intestinal microbial composition through intestinal physiological modifications [7].

The increased bacterial richness that we have observed in our HT patients might be related to the bacterial overgrowth in the intestinal tract of these hypothyroid patients [25,26]. On the other hand, our GD patients showed a lower evenness in their microbiota populations in comparison to the HDs, which could indicate that in this population, could be dominant features. However, biodiversity did not show any significant differences among our study groups, which was aligned with reported works [15,27,28].

Our core microbiome results have demonstrated that the identified features at family and genus levels were mainly shared by our three study groups. However, despite this apparent normality, we have found some specific bacteria characteristic of each particular condition.

Taking together the differences in the feature abundances of each group and their respective core microbiomes, we have found important trends associated with each study group. About GD patients, we found a significant increase in the *Fusobacteriaceae* family and its *Fusobacterium* genus in GD. *Fusobacterium* is a well-recognized pro-inflammatory bacterium [29] and *Fusobacterium nucleatum* has been already reported in GD patients [30]. *Sutterella* was found higher in abundance in GD patients than in HDs. *Sutterella* has been positively related to prediabetes and inflammatory bowel disease [31,32] and it has an immunomodulatory role and pro-inflammatory capacity in the human gastrointestinal tract [33]. Moreover, we have also found a remarkable relationship between *Prevotellaceae* family and *Prevotella* genus with GD group through the core microbiome analysis. *Prevotella* has been linked with GD patients [27] and other autoimmune diseases like rheumatoid arthritis [34]. Thus, *Fusobacterium*, *Sutterella* and *Prevotella*, features relative to GD disease, have been linked to processes related to inflammation and autoimmunity.

In the case of HT patients, the family *Victivallaceae* is increased and is part of their core microbiome. Nevertheless, the role of these bacteria is little known, only some reports linking *Victivallaceae* with diet and obesity [35]. Another member of the core microbiome is the family *Streptococcaceae* and its genus *Streptococcus* that in combination with *Rikenellaceae*, which has been found increased in the HT patients concerning GD, have been reported to take part in the decrease in alpha-diversity in type 1 diabetes, with the capacity to behave as pathogens [36]. This is also the case of *Haemophilus*, which most of its species are considered pathobionts. *H. influenzae* has been reported to induce autoimmune disease by molecular mimicry of an epitope [37]. Furthermore, other core microbiome features of HT like *Alistipes*, *Anaerostipes* or *Dorea* could also be treated as pathobionts that within the disease environment could develop the capacity to damage the host. Together these results suggest that it could be possible that with a larger study these pathobionts from the core microbiome of the tested groups could reach a statistical significance between them.

About HDs related to AITDs, the core microbiome launched that *Christensenellaceae* was characteristic of this group. This result is compatible with studies that identify *Christensenellaceae* family as an important player in human health, associated with human longevity [38] and with lower levels of BMI [39]. As in the other groups, maybe a longer study could trigger differences in the abundance of *Christensenellaceae* with respect to the AITDs. Moreover, our study showed that *Faecalibacterium* genus decreases in autoimmune thyroid conditions compared to HDs, which is aligned with previous works [16,27]. It is important to remark that, although HDs were appropriate for the comparison with the other groups attending to their anthropometric and biochemical variables (no differences were observed with respect to the tested groups), the election of volunteers with a lower BMI could be beneficial for greater differences with respect to the tested groups. This fact could be also considered about the election of volunteers without TPO-Ab, although it is known that low levels are not relevant for the clinical practice if they are not accompanied by other variables [40]. These issues will be considered in future studies.

According to the PICRUSt analysis, GD disease is characterized by enrichment in xenobiotics degradation and metabolism as well as the metabolism of cofactors and vitamins in comparison to HDs. This could be related to the fact that GD patients are characterized by hypermetabolism [41] and could be translated into their microbiota profiles. Moreover, the enrichment in xenobiotics degradation could be related to the fact of these patients are medicated. This result could be indicative of some kind of intervention of the gut microbiota in the medication action, as it has been reported with other drugs [42].

Further analysis was performed to establish a possible relationship between autoimmunity parameters and specific features. Our analyses showed a positive correlation of TSI-ab with *Pasteurellaceae* family shared by AITDs patients. *Pasteurellaceae* family has been previously correlated with GD patients [27]. Thus, our correlation studies showed that some members of the gut microbiota were widely correlated with autoimmunity parameters, indicating that the gut microbiota might be closely related to the AITDs. Our findings may support future research on the interaction of the gut microbiota to the development of the AITDs. Likewise, a prediction model evaluated a possible relationship between predominant concrete bacteria in the AITDs. An unclassified genus of the *Ruminococcaeae* family and *Sutterella* were related to HT patients and GD patients, increasing the risk of presenting positive TPO-ab and TSI-ab, respectively. On one hand, *Ruminococcaceae* has been found as a part of the shared core microbiome of our volunteers [43]. On the other hand, we have already shown the implication of *Sutterella* in inflammatory conditions.

The most promising feature seems to be *Faecalibacterium*. *Faecalibacterium* has been related to a decrease of 94.1% in TSI immunity. In addition, inflammatory processes like inflammatory bowel diseases and colorectal cancer are favored when *Faecalibacterium* is decreased [44,45]. Moreover, in our study, *Faecalibacterium* has been shown as a protective factor, reducing the probability of presenting TSI-ab related to GD patients. The decrease of *Faecalibacterium* could indicate a real dysbiosis in these patients.

Although this study has several strengths, as the complete characterization of the microbiota, several limitations to this research must be acknowledged, like the low number of subjects because of the pilot study nature of our investigation. In this manner, this fact has been advertized throughout the document. Although further longitudinal investigations are necessary to evaluate the progression of the AITDs, these results contribute to the increase in scarce knowledge about the relationship between the gut microbiota and AITDs.

## 5. Conclusions

In this pilot study, our observations demonstrated a gut dysbiosis in AITD patients, may be able to contribute to thyroid disease development. Thus, gut dysbiosis might be related to the immune system development in AITD patients and the loss of tolerance to self-antigens including thyroglobulin and the autoimmunity that triggers AITD. Even though the studies of microbiome and their association to predict disease states are useful for personalized medicine, a deeper understanding of the microbiome might be necessary for the development of evidence-based microbial therapeutics in AITDs. Furthermore, studies with a greater number of subjects and longitudinal studies investigations might be required to evaluate the progression of the AITDs. Nevertheless, our results contribute to the increase in scarce knowledge about the relationship between the gut microbiota and AITDs.

## Figures and Tables

**Table 1:** Clinical and demographic characteristics of patients and healthy donors.

Parameters	HT Patients (*n* = 9)	GD Patients (*n* = 9)	HDs (*n* = 11)	*p*-Value
Sex (M/F, % F)	(0/10, 100)	(2/7, 77.8)	(4/7, 63.5)	0.113
Age (years, mean ± SD)	40.3 ± 9.6	46.2 ± 8.6	48.8 ± 6.2	0.062
Smokers (%)	30	44.4	-----	0.515
Family history of Thyroid disease (%)	50	33.3	-----	0.484
Time of evolution of thyroid disease (months, mean ± SD)	134.4 ± 101.3	16.4 ± 22.6	-----	0.001 *
Anthropometry				
Weight (kg, mean ± SD)	63.4 ± 11.7	66.6 ± 13.0	69.5 ± 8.1	0.354
BMI (kg/m^2^, mean ± SD)	24.9 ± 5.8	25.2 ± 4.7	25.0 ± 2.0	0.831
Waist c. (cm, mean ± SD)	81.4 ± 13.8	86.1 ± 10.9	87.9 ± 8.4	0.321
Hip c. (cm, mean ± SD)	98.3 ± 10.7	101.6 ± 11.3	97.0 ± 4.1	0.391
Blood pressure				
Systolic (mmHg, mean ± SD)	121.6 ± 11.3	119.6 ± 16.2	128.8 ± 17.3	0.636
Diastolic (mmHg, mean ± SD)	75.3 ± 5.1	75.0 ± 15.4	78.6 ± 8.7	0.449
Analytical metabolic				
Glucose (mg/dL, mean ± SD)	86.6 ± 6.5	93.4 ± 4.1	91.0 ± 8.3	0.078
HbA1c (%, mean ± SD)	5.1 ± 0.3	5.3 ± 0.4	5.3 ± 0.3	0.29
Total-C (mg/dL, mean ± SD)	178.5 ± 44.7	192.2 ± 29.6	192.4 ± 47.8	0.568
LDL-c (mg/dL, mean ± SD)	107.7 ± 39.1	109.4 ± 10.2	110.1 ± 38.1	0.758
HDL-c (mg/dL, mean ± SD)	56.8 ± 11.7	65.6 ± 22.2	60.5 ± 12.9	0.666
TGs (mg/dL, mean ± SD)	70.1 ± 16.6	85.2 ± 31.9	108.8 ± 59.0	0.132
CRP (mg/dL, mean ± SD)	3.1 ± 0.0	3.2 ± 0.4	4.1 ± 1.4	0.116
Thyroid profile				
TSH (µIU/mL, mean ± SD)	2.6 ± 2.8	3.3 ± 8.5	2.2 ± 1.0	0.030 *
FT4 (pmol/L, mean ± SD)	15.4 ± 2.2	15.2 ± 3.1	15.2 ± 1.3	0.76
FT3 (pmol/L, mean ± SD)	3.8 ± 0.2	5.5 ± 2.3	4.8 ± 0.4	0.002 *
TPO-Ab (IU/mL, mean ± SD)	1186.7 ± 358.4	792.0 ± 621.7	160.3 ± 381.3	0.001 *
TPO-Ab > 60 IU/mL (P/N, % P)	(10/0, 100)	(6/3, 66.7)	(2/9, 18.2)	0.000 *
TSI-Ab (IU/mL, mean ± SD)	3.5 ± 7.2	16.8 ± 34.1	0.8 ± 0.1	0.000 *
TSI-Ab > 2 IU/mL (P/N, % P)	(1/9, 10)	(9/0, 100)	(0/11, 0)	0.000 *

BMI, body mass index; CRP, C-reactive protein; FT3, free triiodothyronine; FT4, free thyroxine; GD, Graves–Basedow’s disease; HDs, healthy donors; HDL-c, high-density lipoprotein; Hip c. hip circumference; HT, Hashimoto’s thyroiditis; LDL-c, low-density lipoprotein cholesterol; M/F, male/female; P/N, positive/negative ratio; TGs, triglycerides; Total-C, total cholesterol; TPO-Ab, thyroperoxidase antibody; TSI-Ab thyroid stimulating immunoglobulin antibody; Waist c. waist circumference. * Significant differences *p* < 0.05.

**Table 2:** Simple linear correlation relationship of autoimmunity and thyroid profile with gut microbiota (taxa abundances: samples at phylum, family, and genus levels).

Plylum Level	Family or Genus Level	TPO-Ab	TSI-Ab
Bacteroidetes		-----	-----
	*Alistipes*	*r* = 0.432; *p* = 0.019	-----
Firmicutes		-----	-----
	*Lactobacillaceae*	-----	*r* = 0.517; *p* = 0.006
	*Lactobacillus*	-----	*r* = 0.517; *p* = 0.006
	*Faecalibacterium*	*r* = −0.453; *p* = 0.014	*r* = −0.406; *p* = 0.036
	*Ruminococcaceae unclasificated*	*r* = 0.408; *p* = 0.028	-----
Proteobacteria		-----	-----
	*Enterobacteriaceae*	*r* = 0.416; *p* = 0.025	-----
	*Pasteurellaceae*	-----	*r* = 0.441; *p* = 0.021

TPO-Ab, thyroperoxidase antibody; TSI-Ab, thyroid stimulating immunoglobulin antibody.

**Table 3:** Adjusted model for autoimmunity thyroid profile (Positive TPO-Ab >60 IU/mL)—Gut microbiota community.

Positive TPO Autoimmunity (TPO-Ab >60 IU/mL)		
	*Ruminococcaeae unclassified*	
	OR (CI)	*p*
Crude model	1.494 (1.041–2.145)	0.029
Model 1	1.474 (1.026–2.413)	0.038

Binary logistic regression analysis: Odds ratio (OR) and 95% confidence interval (CI) for the association between positive thyroid autoimmunity and gut microbiota. Positive levels of TPO antibody were defined as a level >60 IU/mL. Dependent variable: positive TPO autoimmunity—TPO-Ab levels <60 IU/mL (0) vs. TPO-Ab levels >60 IU/mL (1). Model 1: adjusted for sex, age, and BMI.

**Table 4:** Adjusted model for autoimmunity thyroid profile (Positive TSI-Ab >2 IU/mL)—Gut microbiota community.

Positive TSI Autoimmunity (TSI-Ab >2 IU/mL)				
	*Sutterella*	*Faecalibacterium*		
	OR (CI)	*p*	OR (CI)	*p*
Crude model	1.351 (1.053–1.734)	0.018	0.059 (0.004–0.958)	0.047
Model 1	1.499 (1.041–2.158)	0.030	0.025 (0.001–0.900)	0.044

Binary logistic regression analysis: ddds ratio (OR) and 95% confidence interval (CI) for the association between positive thyroid autoimmunity and gut microbiota. Positive levels of TSI antibody were defined as a level >2 IU/mL. Dependent variable: positive TSI autoimmunity—TSI-Ab levels <2 IU/mL (0) vs. TSI-Ab levels >2 IU/mL (1). Model 1: adjusted for sex, age, and BMI.

## Supplementary Materials

The following are available online at https://www.mdpi.com/2075-4426/10/4/192/s1, Table S1: Shared families and genera from each core microbiomes of the study group. Figure S1: Clustering of fecal bacterial communities according to the different study groups by PCoA using unweighted and weighted UniFrac distances. Figure S2: Significant differences in predicted functional composition at the level 3 of KEGG Pathways of the gut microbiota among HDs and GD patients.
