# Supplementary material for: Differential Microbial Pattern Description in Subjects with Autoimmune-Based Thyroid Diseases: A Pilot Study
Source: J Pers Med. 2020 Oct 26;10(4):192. doi: 10.3390/jpm10040192 (PMC7712884; doi:10.3390/jpm10040192)
Supplement: Supplementary file 1 [file jpm-10-00192-s001.pdf]

Differential microbial pattern description in subjects with autoimmune-based thyroid diseases

Isabel Cornejo-Pareja, Patricia Ruiz-Limón, Ana María Gómez-Pérez, María Molina-Vega, Isabel Moreno-Indias, Francisco José Tinahones.

Supplementary Materials

Table S1. Shared families and genera from each core microbiomes of the study group.

**15 common families in HT patients, GD patients and HDs**
Bacteroidaceae
Lachnospiraceae
Ruminococcaceae
Alcaligenaceae
Desulfovibrionaceae
Rikenellaceae
Porphymonadaceae
Odoribacteriaceae
Erysipelotrichaceae
Barnesiellaceae
Coriobacteriaceae
Veillonellaceae
Enterobacteriaceae
Bifidobacteriaceae
Clostridiaceae
**12 common genera in HT patients, GD patients and HDs**
Bacteroides
Sutterella
Parabacteroides
Bilophila
Ruminoccocus
Odoribacter
Oscillospira
Faecalibacterium
Blautia
Lachnospira
Bifidobacterium
Coprococcus

GD, Graves-Basedow´s disease; HDs, healthy donors; HT, Hashimoto’s thyroiditis.

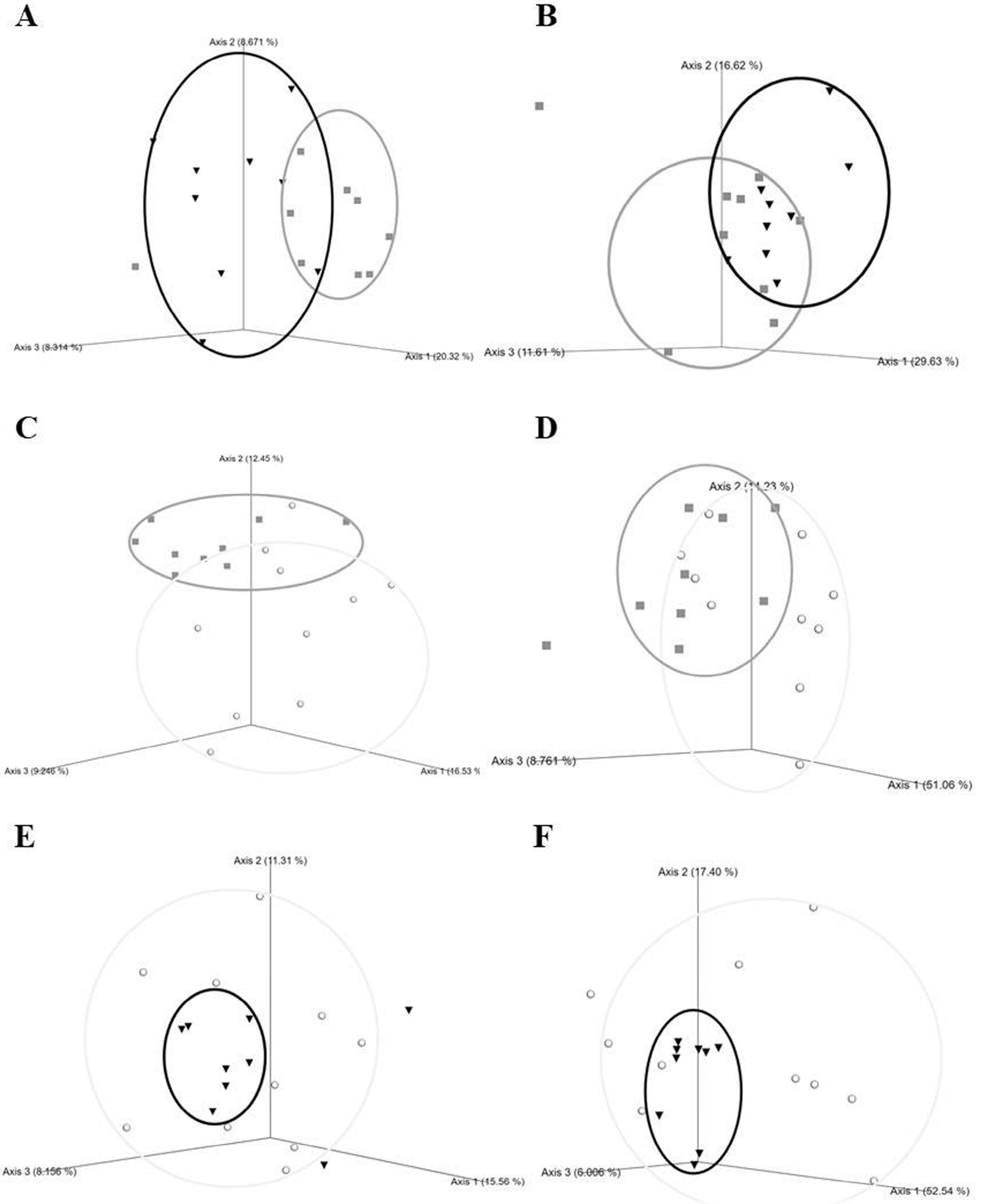

Figure S1. Clustering of fecal bacterial communities according to the different study groups by PCoA using unweighted and weighted UniFrac distances. **(A)** Unweighted UniFrac distances between both AITDs patients, p= 0.115 and **(B)** Weighted UniFrac distances between AITD patients, p= 0.169. **(C)** Unweighted UniFrac distances between GD patients and HDs, p= 0.005 and **(D)** Weighted UniFrac distances between GD patients and HDs, p= 0.007. **(E)** Unweighted UniFrac distances between HT patients and HDs, p= 0.05 and **(F)** Weighted UniFrac distances between HT patients and HDs, p= 0.021. Circles belong to the HDs; squares to GD patients and triangles to HT patients.

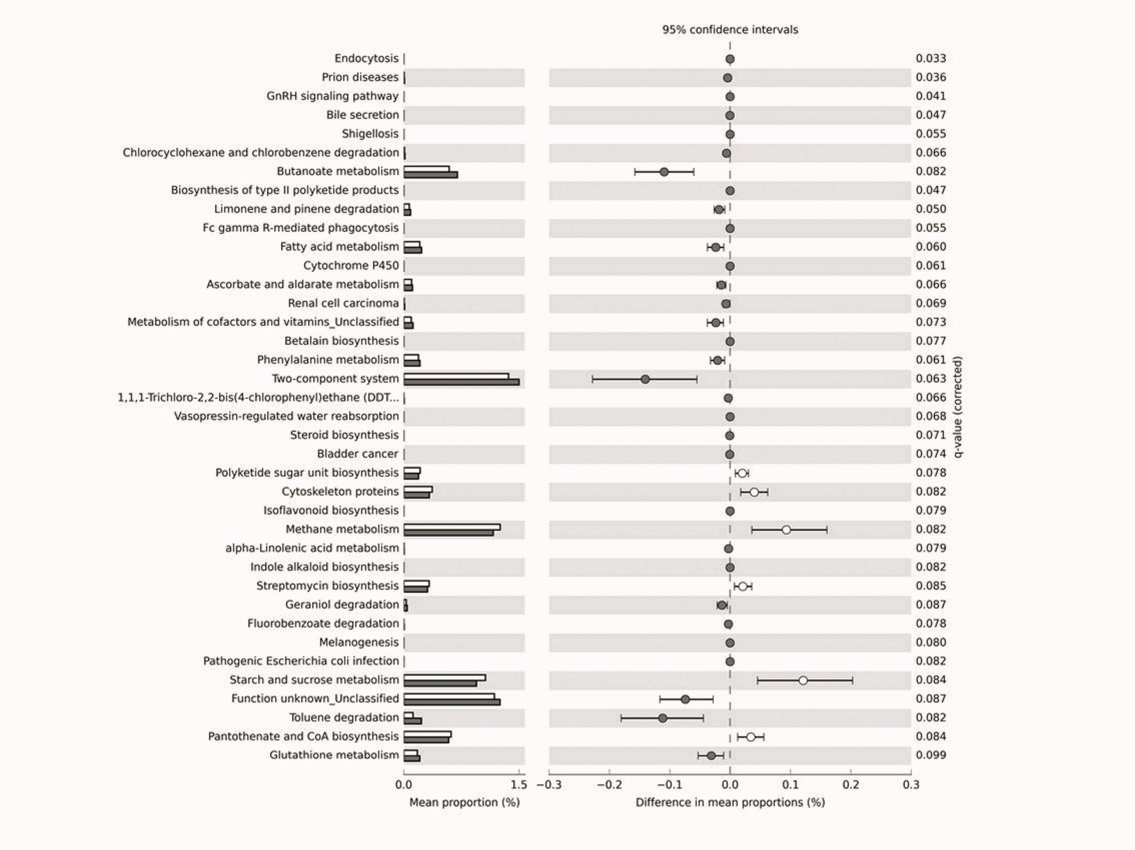

Figure S2. Significant differences in predicted functional composition at the level 3 of KEGG Pathways of the gut microbiota among HDs (white) and GD patients (dark grey). Only functional capacities with p<0.1 are shown; q=p-value FDR corrected.
